# Probiotic modulation of the microbiota-gut-brain axis and behaviour in zebrafish

**DOI:** 10.1038/srep30046

**Published:** 2016-07-15

**Authors:** Luca Borrelli, Serena Aceto, Claudio Agnisola, Sofia De Paolo, Ludovico Dipineto, Roman M. Stilling, Timothy G. Dinan, John F. Cryan, Lucia F. Menna, Alessandro Fioretti

**Affiliations:** 1 Department of Veterinary Medicine and Animal Productions, University of Naples Federico II, Napoli, Italy; 2 Department of Biology, University of Naples Federico II, Napoli, Italy; 3 APC Microbiome Institute, University College Cork, Cork, Ireland; 4 Department of Psychiatry and Neurobehavioural Science, University College Cork, Cork, Ireland; 5 Department of Anatomy and Neuroscience, University College Cork, Cork, Ireland

## Abstract

The gut microbiota plays a crucial role in the bi-directional gut–brain axis, a communication that integrates the gut and central nervous system (CNS) activities. Animal studies reveal that gut bacteria influence behaviour, Brain-Derived Neurotrophic Factor (BDNF) levels and serotonin metabolism. In the present study, we report for the first time an analysis of the microbiota–gut–brain axis in zebrafish (*Danio rerio*). After 28 days of dietary administration with the probiotic *Lactobacillus rhamnosus* IMC 501, we found differences in shoaling behaviour, brain expression levels of *bdnf* and of genes involved in serotonin signalling/metabolism between control and treated zebrafish group. In addition, in microbiota we found a significant increase of Firmicutes and a trending reduction of Proteobacteria. This study demonstrates that selected microbes can be used to modulate endogenous neuroactive molecules in zebrafish.

There is an emerging understanding of the bi-directional crosstalk governing gut-to-brain communication in health and disease of both organs. Accordingly, not only the brain can affect gut functions, but the gut can also induce changes in the central nervous system (CNS) and there is now compelling evidence for various links between the enteric microbiota and brain function^1^,^2^,^3^. This connection is becoming increasingly relevant in novel therapeutic strategies to target psychiatric disorders such as depression and anxiety disorders. As such, it has been shown that absence or modification of the enteric microbiota affects stress-associated anxiety-like and depressive-like behaviours^4^,^5^. This has lead to the postulation of psychobiotics, i.e. live microorganisms that have beneficial psychotropic effects on the host^6^. To date, multiple probiotic bacteria with psychotropic potential have been identified, including strains of the genera *Bifidiobacterium*^7^,^8^,^9^,^10^, *Lactobacillus*^9^,^10^,^11^,^12^ and *Enterococcus*^13^. A very promising potential psychobiotic with demonstrated effectivity in mice and humans is *Lactobacillus rhamnosus*^11^,^12^.

Importantly, the microbiota has been implicated in altering neurotrophic factors^7^,^8^,^14^,^15^,^16^,^17^, a class of proteins playing roles in controlling neuronal function and maintaining cellular integrity, survival, differentiation, and synaptic plasticity^18^,^19^,^20^. Among all neurotrophins, Brain Derived Neurotrophic Factor (BDNF) is the most well conserved throughout vertebrate evolution^1^,^21^,^22^. As such, the primary amino acid sequences of zebrafish (*Danio rerio*) and human BDNF are 91% identical^22^,^23^,^24^. While the distribution pattern of *bdnf* mRNA and protein has been described in the CNS of rat^25^, mouse^26^, human^27^ and zebrafish^28^, BDNF has also been observed in other organs and tissues of adult and developing zebrafish^29^. Changes in BDNF signalling is relevant to a range of human neuronal and psychiatric disorders^30^, as well as to biological systems involved in the stress response^31^.

Using rodents as model organisms, multiple studies have also implicated the microbiota in regulation of serotonin signalling and metabolism^16^,^32^,^33^. The monoamine serotonin, or 5-hydroxytryptamine (5-HT), is one of the primary neurotransmitters modulating physiological and behavioural processes in the CNS^34^,^35^,^36^ and the serotonergic system is highly conserved in vertebrate species. Also zebrafish *Danio rerio* possesses a complex serotonergic system featuring all major genes for 5-HT synthesis, metabolism and signalling^37^, similar to those observed in humans and rodents^38^. As such, zebrafish possess three copies of the *tph* gene (*tph1a, tph1b and tph2*) encoding tryptophan hydroxylase, the rate-limiting enzyme in serotonin synthesis, two genes encoding serotonin transporters homologous to those of vertebrates, *slc6a4a* and *slc6a4b* (previously *serta* and *sertb*)^34^, and multiple receptors homologs, e.g. *htr1aa*, homologue of the mammalian serotonin receptor 1A, and *htr1ab*, specific of fishes. Zebrafish also have one functional monoamine oxidase gene (*mao*) exhibiting a strong affinity profile for serotonin^37^,^39^. Zebrafish is a well-known model organism, increasingly used in translational neuroscience and behavioural research^40^. Interestingly, it is a highly social species and shoaling is a typical group forming behaviour often seen in cyprinids (Cyprinidae). Shoaling behaviour has been observed both in nature^41^ and in the laboratory^42^,^43^ as one of the most robust and consistent behavioural features of this species^40^.

To explore probiotics as potential therapeutic psychotropic agents in psychiatric diseases and to further highlight the use of the zebrafish model to describe the impact of psychobiotics on brain function and behaviour, we here assessed the effects of the probiotic strain *Lactobacillus rhamnosus* IMC 501 on shoaling behaviour in healthy, wild-type zebrafish. This strain was already used in zebrafish, where it generates beneficial effects such as reduction of hepatic cholesterol level, improved adsorption of nutrients and increased production of short-chain fatty acids^44^. Moreover, we investigate central and peripheral expression levels of the *bdnf* gene and of the serotonergic genes *tph1a*, *tph1b*, *tph2*, *htr1aa*, *slc6a4a* and *mao*, in brain and gut of zebrafish. We also evaluated the microbiota profile by using 16S rRNA sequencing after probiotic administration.

## Results

### L. rhamnosus administration changes shoaling behaviour

**Figure 1:**
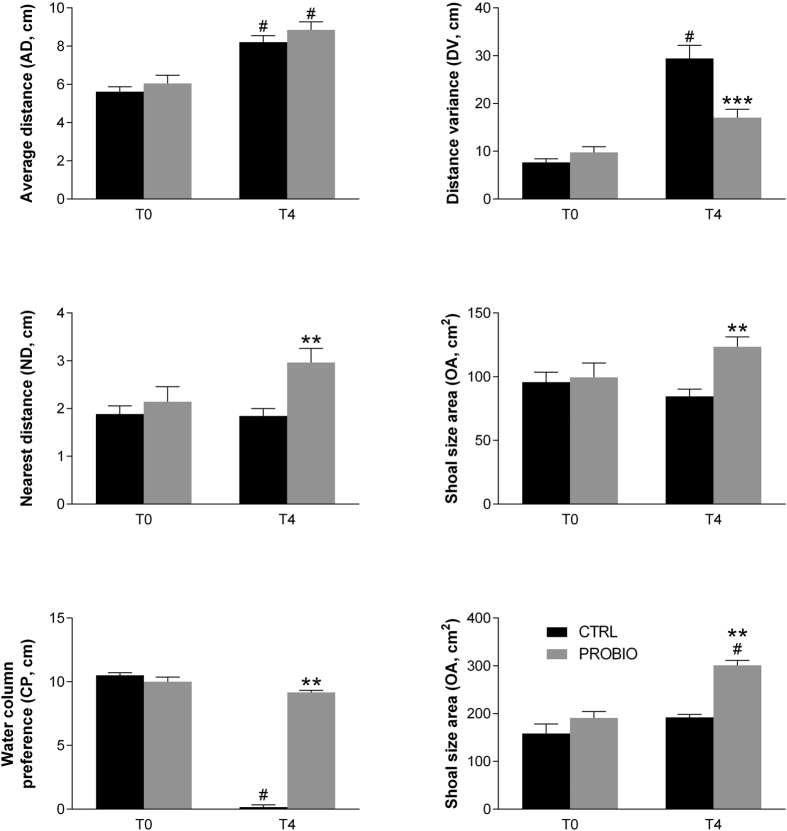
Probiotic treatment alters zebrafish shoaling behaviour. Average Distance (AD), Distance Variance (DV), Nearest Distance (ND), Occupied Area (OA), Column Preference (CP) and Shuttling Frequency (SF) measurements (Supplementary Figure 1) at the beginning (T0) and end of 4 weeks (T4) of probiotic treatment, are shown from both control (CTRL) and treated (PROBIO) animals. Bars represent SEM. ^#^indicates significant difference from T0 (Two-way ANOVA with Sidak’s post-hoc test, p < 0.05); asterisks indicate statistical significant difference between the CTRL and PROBIO groups (Two-way ANOVA with Sidak’s post-hoc test: ***p < 0.001, **p < 0.01, *p < 0.05). DV F_interaction_ = 17.79, p < 0.0001; ND F_treatment_ = 7.948, p = 0.0059; OA F_interaction_ = 4.423, p = 0.0483; CP Finteraction = 377.8, p < 0.0001; SF F_interaction_ = 7.862, p = 0.0127.

Zebrafish shoaling behaviour was analysed for Average Distance (AD), Distance Variance (DV), Nearest Distance (ND), Occupied Area (OA), Column Preference (CP) and Shuttling Frequency (SF) at day 0 and after 4 weeks in both control (CTRL) and probiotic treated (PROBIO) groups. Control group (CTRL) displayed behavioural adaptation to the tank during the 4 weeks of treatment (Fig. 1). In particular, AD and DV significantly increased, while CP significantly decreased. As shoal size area (OA) did not change, these data indicate that after adaptation animals were less uniformly distributed in the shoal, and tended to spend most of their time in the upper part of the tank. Shoal cohesion of zebrafish fed with probiotics (PROBIO) was significantly different from controls (CTRL) (Fig. 1). Although AD was not significantly different between the two groups for treatment, DV (F_interaction_ = 17.79, p < 0.0001) and ND (F_treatment_ = 7.948, p = 0.0059) showed significant dependence on treatment and time (Table S1). OA, was also significantly increased in the PROBIO group (F_interaction_ = 4.423, p = 0.0483). The two groups showed completely different preferences for location in the water column (CP) (F_interaction_ = 377.8, p < 0.0001) (Table S1). More importantly, SF (F_interaction_ = 7.862, p = 0.0127) was strongly increased in the PROBIO group. Together, these data suggest that feeding of the probiotic *L. rhamnosus* IMC 501 significantly alters social and explorative behaviour in zebrafish (Video S1).

### Probiotic administration modulates the gut microbial communities

**Figure 2:**
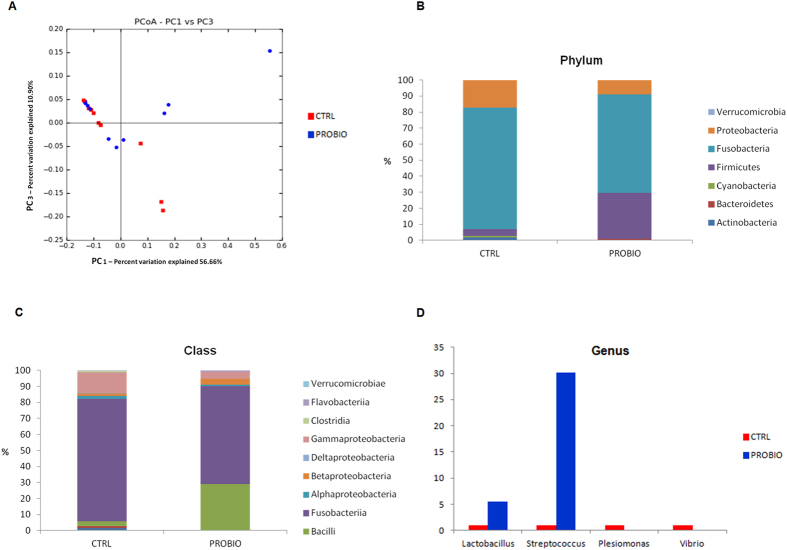
Whole intestine bacterial community analysis of adult zebrafish after probiotic treatment. (**A**) Principal coordinates analysis (PCoA) plots using weighted UnicFrac distances. Stacked bar chart representing the relative abundance of bacterial phylum (**B**) and classes (**C**). (**D**) Fold change of the genus *Lactobacillus*, *Streptococcus*, *Plesiomonas* and *Vibrio* in the PROBIO relative to the CTRL group.

The α-diversity indices Chao1 (138.02 ± 40.36 CTRL, 133.25 ± 30.00 PROBIO) and Shannon (4.12 ± 0.59 CTRL, 3.92 ± 0.97 PROBIO) of the gut microbiota in CTRL and PROBIO zebrafish were not significantly different, indicating that the dietary administration of *L. rhamnosus* does not affect overall richness of the zebrafish gut bacterial community. Similarly, weighted two-dimensional Principal Coordinates Analysis showed that PROBIO samples largely clustered with CTRL samples, demonstrating overall similarity of the gut bacterial communities between the two groups (Fig. 2A). However, we noticed differences at the phylum and class levels (Fig. 2B,C). Fusobacteria dominated both groups, accounting for 75.83% (CTRL) and 61.18% (PROBIO) of the OTUs retrieved. Proteobacteria (17.24% CTRL, 9.01% PROBIO), Firmicutes (4.10% CTRL, 28.75% PROBIO), and Cyanobacteria (0.97% CTRL, 0.19% PROBIO) were also present in both groups, yet at different abundances. Actinobacteria were present only in the CTRL (1,86%) and Bacteroidetes (0.78%) only in the PROBIO samples. The abundance of *Lactobacillus* (8,3%) in the PROBIO group is likely representative of the dietary administration of *L. rhamnosus* (Phylum: Firmicutes, Class: Bacilli), suggesting that the fed probiotic bacteria colonised the gut, in agreement with previous studies^45^,^46^,^47^. In addition, in the PROBIO group there is a significant increase of the *Streptococcus* genus (t-test p value 0.025, Table S2; Fig. 2D). The abundance of both *Lactobacillus* and *Streptococcus* might be related to the symbiotic relationship between *L. rhamnosus* and *Streptococcus thermophilus* described in zebrafish after probiotic administration44,^46^,^47^. On average, Proteobacteria, that include potentially pathogenic genera (e.g. *Vibrio* and *Plesiomonas*, Fig. 2D), were less represented in the PROBIO group (9.01%) than in the CTRL (17.84%), although this difference was not statistically significant.

### Probiotic feeding induces changes in neuronal gene expression in brain and gut

**Figure 3:**
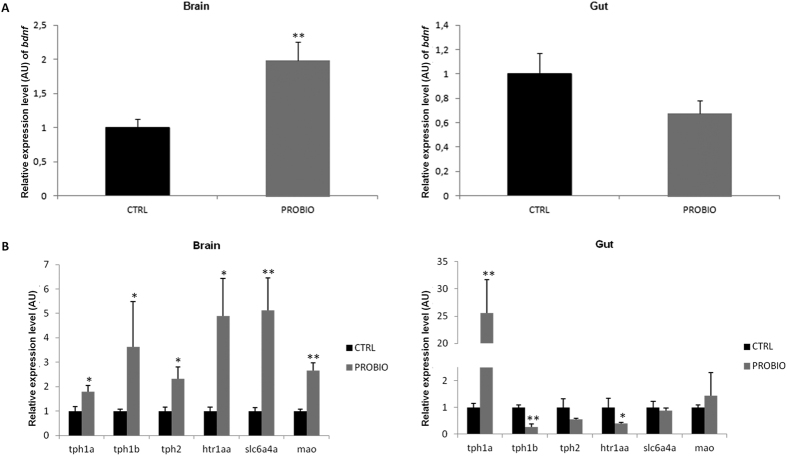
Probiotic feeding induces changes in neuronal gene expression in brain and gut. Relative expression of the *D. rerio bdnf* (**A**) and *tph1a*, *tph1b*, *tph2*, *htr1aa*, *slc6a4a, mao* (**B**) genes in brain and gut of zebrafish treated with *L. rhamnosus* (PROBIO) and controls (CTRL). The mean relative expression levels of the PROBIO group are normalized to the mean relative expression of the CTRL group. AU, arbitrary units. Bars represent SEM. Asterisks indicate statistical significant difference between the CTRL and PROBIO groups: **t-test p < 0.01, *t-test p < 0.05.

Previous studies on the gut-brain axis in rodent models have repeatedly shown altered expression of *bdnf* in certain brain regions under various conditions^7^,^8^,^14^,^15^,^17^. In addition, BDNF signalling is known to be involved in social behaviour^48^,^49^. As such, the expression of *bdnf* in the brains of rodent models is very plastic and regulated in a dynamic way. A very well-studied molecular pathway resulting in elevated levels of *bdnf* mRNA is the CREB (cAMP response element binding protein) signalling pathway that gets activated upon neuronal excitation. Many behavioural paradigms have been shown to induce *bdnf* expression in various brain regions including novelty exposure (e.g. social novelty, novel environment, environmental enrichment) and learning tasks. On the other hand, a reduction in *bdnf* levels is often a signature of reduced cognitive abilities. Importantly, reduced levels of *bdnf* have been associated with depression and it has been suggested that conventional antidepressants and ketamine mediate their antidepressant-like effects by increasing *bdnf* in particular in the hippocampus, making it an essential determinant of antidepressant efficacy^50^. We therefore determined if the expression of *bdnf* was altered after probiotic administration in zebrafish. In the brain, the PROBIO group showed a significant (near two-fold) increase of *bdnf* expression compared to the CTRL group (t-test p value 0.001) (Fig. 3A). In contrast, we did not detect any significant difference between the PROBIO and CTRL groups in the gut, although there was a trend towards decreased expression.

Furthermore, socio-emotional behaviour in vertebrates is heavily dependent on the serotonergic system^51^,^52^. Moreover, microbial influence on the serotonergic system has been proposed as a key mediator of gut-brain signalling^16^,^33^. Therefore, we next investigated potential changes in expression of selected genes involved in serotonin signalling and metabolism (t*ph1a, tph1b, tph2, htr1aa, slc6a4a* and *mao*), both in the gut and in the brain of zebrafish fed with *L. rhamnosus* IMC 501 (Fig. 3B). In the brain, five genes showed a statistically significant increase of the relative expression levels in the PROBIO group (t-test p values: t*ph1a* 0.03, *tph2* 0.01*, htr1aa* 0.01*, slc6a4a* 0.004 and *mao* 0.0003), indicating that the probiotic administration changes the expression levels of genes involved in the serotonergic system. In contrast, in the gut, we detected a reduction of the expression levels for the genes *tph1a* (t-test p value 0.001)*, tph1b* (p = 0.001) and *htr1aa* (p = 0.03), while the other genes did not show significant differences between the PROBIO and CTRL groups. Together, these data suggest tissue-specific modulation of the serotonergic system.

## Discussion

Zebrafish is an increasingly important model organism in neuroscientific basic and translational research^53^. Zebrafish digestive tract is similar to that of mammals in its development, organisation and function and zebrafish are well suited for studying host-microbe interactions as they have innate and adaptive immune systems similar to higher vertebrates^54^. Surprisingly, to our knowledge, there have been no studies yet investigating the influence of beneficial or commensal gut microbes on the gut-brain axis in this model organism. Yet, host-microbe interactions in the gut are now widely accepted to affect brain function and behaviour and considered as a potential target in psychiatric diseases^55^. We have therefore decided to explore the effects of feeding healthy, wild-type zebrafish with the probiotic strain *L. rhamnosus* IMC 501 on shoaling behaviour, a measure of basal exploratory and social behaviour.

In our study the shoaling pattern of zebrafish differed significantly from controls after 4 weeks of probiotic treatment. In particular, the probiotic treated group was more uniform and showed more homogeneity within the shoal as evidenced by a decreased average distance variance (DV). In addition, the nearest distance (ND) and occupied shoal area (OA) were significantly increased after treatment. Concerning the water column preference (CP), the PROBIO group showed a completely different behavioural pattern. While the control group changed significantly to prefer the upper parts of the water tank at the end of the 4 weeks of observation, which is likely due to habituation to the aquarium, the probiotic treated group continued to prefer the medium/deeper part of the tank. Furthermore, a significant increase in shuttling frequency (SF) indicates a strong increase in locomotor activity. These data indicate an increased exploratory behaviour in the PROBIO animals^56^. The different behaviour between CTRL and PROBIO group suggests a higher exploration capability and attention/alert in the PROBIO group.

Notably, the detected pronounced changes in behaviour were accompanied by strongly increased expression levels of *bdnf* and several genes involved in serotonin signalling metabolism in the brain, while their expression in the gut was reduced. In fact, BDNF and the serotonergic system in the brain work synergistically and influence each other mutually on the transcriptional level, via epigenetic mechanisms, and by direct protective and regenerative effects of BDNF on serotonergic neurons throughout development and in later life^57^.

Most well established, the serotonergic system is linked to many behavioural traits^58^. As such, selective serotonin reuptake-inhibitors (SSRIs) are the first-line treatment in severe depression and also recommended to treat generalized anxiety disorder^59^. Increased serotonin production and receptor expression in the zebrafish brain is therefore perfectly in line with the observed behavioural change induced by *L. rhamnosus* administration. In fact, both, the serotonergic system as well as BDNF levels, are well established effectors of the microbiota in the host. As such, Neufeld *et al*.^60^ found decreased expression of the serotonin receptor 1a (*Htr1a*) in germ-free mice^60^. In addition, a recent study showed increased levels of serotonin in the hippocampus of germ-free mice^16^, likely due to increased tryptophan uptake in the absence of tryptophan-metabolising microorganisms in the gut^16^. This study also revealed altered BDNF levels, which has now commonly been found in animal models with altered microbiota^7^,^8^,^14^,^16^. Moreover, the microbiota is directly involved in stimulating serotonin release from enterochromaffin cells in the gut epithelium, regulating enteric nervous system and vagus nerve activity^33^. Thus, our findings support existing literature connecting the microbiota with these systems also in the zebrafish.

Of note, we found different expression of *bdnf* and serotonin-related genes in the gut and the brain.

Recent findings show that BDNF plays an important role in gastrointestinal function and an increased level of BDNF in the colon of diarrhea-predominant irritable bowel syndrome (IBS) patients with abdominal pain was described^61^. However, the regulation of the expression of *bdnf* in gut is still unclear. Additionally, serotonin release in the gut and the enteric nervous system is regulated independently of regulation in the CNS^62^. It is therefore likely that *L. rhamnosus* supplementation has independent effects on these two systems, which does not preclude the possibility that both systems directly or indirectly contribute to the observed change in shoaling behaviour.

Analysis of the microbiota composition revealed that Proteobacteria, Fusobacteria and Firmicutes appeared consistently in the gut microbiota of zebrafish. Proteobacteria and Fusobacteria are common members of the gut microbiota in adult zebrafish and are especially well adapted to conditions of the fish gastro-intestinal tract in a surrounding aquatic environment^63^. The Firmicutes phylum (including *Streptococcus* spp. and *Lactobacillus* spp.) increased significantly in PROBIO group (t-test p value 0.038, Table S2) at the expense of representatives of the Proteobacteria fraction (including *Vibrio* spp. and *Plesiomonas* spp.) (Fig. 2D). This result is in agreement with previous studies reporting increased abundance of *Lactobacillus* and *Streptococcus* in zebrafish after probiotic administration, resulting in several beneficial effects^44^,^46^,^47^. Interestingly, a higher proportion of Proteobacteria, together with a lower abundance of Firmicutes, characterizes the intestinal microbiota dysbiosis in zebrafish model with inflammatory bowel disease (IBD)-like colitis and correlates significantly with enterocolitis severity, which mirrors changes in the human gut microbiota in IBD^64^.

## Conclusion

So far most findings linking the microbiota with virtually all of the host physiological processes have originated from rodent studies. Compelling and rapidly accumulating evidence highlights the strong impact of the presence and activity of certain microbes in the gut on host physiology and behaviour. Notably, many studies have made use of animals raised in a sterile, germ-free environment. Using this approach it was found that the microbiota is critical for the host’s serotonergic system and tuning the stress response at a behavioural and endocrine level, which has further implications for our perception of stress-related psychiatric and gastrointestinal diseases. While these findings are stimulating, it has to be acknowledged that germ-free animals represent a highly artificial model that is uniquely useful to determine physiological processes influenced by the microbiota in a black-or-white manner, rather than a translatable model for human disease. Taken together, our data reveal specific, so far undescribed behavioural and neurochemical changes in healthy zebrafish, induced by alteration of the enteric microbiota using a potential psychobiotic bacterium (*L. rhamnosus* IMC 501), demonstrating psychotropic effects on the behavioural and molecular level. We may assert that the bidirectional signalling between microbiota and brain exists also in zebrafish, consolidating it as a valuable animal model for translational medicine.

While our data highlight the fact that supplementation with a probiotic is sufficient to induce sizeable effects on at least three levels of readout, the microbiota, neurochemistry and behaviour, further experimentation is necessary to connect these domains in a causal manner. Another limitation of this study, as in most other studies investigating gut-brain communication, is incomplete mechanistic insight into the routes connecting the increased abundance of the probiotic with altered neurochemistry and behaviour. In addition to modulation of the immune system via secreted metabolites and microbial cell wall material, especially differential stimulation of the vagus nerve have been shown to be among the routes of communication that mediates, for example, the antidepressant-like effects of *L. rhamnosus* in mammals^11^.

A deeper understanding of these communication mechanisms will be crucially important for the development of any microbiota-based therapeutic strategy for psychiatric diseases in human and animal health and for the discovery of alternative pathways and substrates to treat brain disorders that do not respond to available drugs.

## Methods

### Ethics Statement

All fish were treated in accordance with the Directive of the European Parliament and of the Council on the Protection of Animals Used for Scientific Purposes (directive 2010/63/EU) and in agreement with the Bioethical Committee of University of Napoli Federico II. All experiments involving fish were approved by the Bioethical Committee of the University of Naples Federico II (authorization protocol number 47339-2013). Following behavioural testing, the animals were euthanized by immersion in overdose 500 mg/ L-1 of 3-aminobenzoic acid ethyl ester (MS-222) buffered to pH 7.4 (Sigma–Aldrich, USA).

### Animals and husbandry

Adult 4–6-month-old male and female zebrafish of heterozygous “wild type” strain were obtained from local commercial distributors (Carmar, Napoli, Italy). All fish were given at least 14 days to acclimate to the laboratory environment and housed in groups of 12 fishes per 30-L tank. All tanks were filled with deionized water before introducing fish. Fish were fed two times daily with sterilized commercial food (Sera Vipagran, Germany). The room and water temperatures were maintained at 25–27 °C. Illumination (1010 ± 88 lx) was provided by ceiling-mounted fluorescent light tubes on a 14-h cycle (D:N = 14h:10h) consistent with the standards of zebrafish care^65^. All fish used in this study were experimentally naïve. Two experimental groups were evaluated: a control group (CTRL, n = 12) and a probiotic-treated group (PROBIO, n = 12). The experiment was repeated twice to obtain biological duplicates.

### Probiotic administration

The control group (CTRL) was fed twice per day with the commercial diet only and the probiotic-treated group (PROBIO) was fed twice per day with the commercial diet and twice per day with the lyophilized probiotic strain *L. rhamnosus* IMC 501, provided by Synbiotec (Camerino, Italy) via rearing water at a final concentration of 10^6^ colony-forming units/g (0,01 g/l), according to the manufactory suggestions, for 28 days. Before the administration of the lyophilized *L. rhamnosus* IMC 501, a viability test was performed (see Supplementary information, Supplementary methods).

### Behavioral testing

Fish swimming behaviour was video-recorded on the first day of probiotic treatment (T0) and at the end of treatment at 28 days (T4). Recording was performed always at the same hour (between 14:00 and 15:00) with a Nikon D7000 camera for 6 min, acquired and analysed with 2D video tracking analysis and modelling tool (Tracker, California, USA) (www.cabrillo.edu/~dbrown/tracker/). In line with Blaser and Gerlai^66^ and Gerlai^40^, the video tracking data were used to determine the following behavioural measures: Average Distance (AD), Distance Variance (DV), Nearest Distance (ND), Occupied Area (or Shoal size area) (OA), Water Column Position (CP) and Shuttling Frequency (SF) (Supplementary Figure 1). In particular, AD represents the inter-individual distance and defines and calculates the average of all distances between a fish and its shoal members. Each fish thus will get an AD value and this value is calculated for all fish within a shoal and for any given moment of time sampled. As it takes the position of all fish in the shoal, AD is a powerful measure of shoal cohesion^40^. DV is the AD variance for each fish. Notably, this variability depends on the relative position of each fish within a shoal, so that its mean value, when calculated for the entire shoal, represents the homogeneity of the distribution of fish within that shoal. The less uniformly the shoal members are distributed, the larger the variance will be. ND calculates the nearest distance of each single fish to its closest neighbour, and represents a measure of the shoal cohesion less powerful than AD, but independent from the shoal size^40^. OA, which also measure shoal cohesion, calculates the occupied area of all animals in a temporal unit on a two-dimensional plane. CP represents the water column position (surface = 0) and indicates the deep preference of shoaling animals in the tank^56^. SF, which measures the locomotor activity of animals in the shoal^66^, is the total number of times that the fish entered the left, the right, the upper, and the lower halves of the tank. For AD, DV and ND measurements each fish was tracked on six frames randomly selected from each video. For SF measurements, five 10 sec intervals randomly selected from each video were analysed. For OA and CP analysis, ImageJ 1.49 software (National Institute of Mental Health, Bethesda, Maryland, USA) was used to select the areas occupied and the deep position of each fish in the water column in all video collected. Here, ten images were randomly selected from each video, one every twenty seconds.

Behaviour was evaluated by two independent, trained observers (intra-rater reliability > 0.90). Results are reported as means ± SEM. Two-way ANOVA with Sidak’s post-hoc test was used to evaluate the significance of the effect of the probiotic treatment, using GraphPad Prism version 6.00 for Windows (GraphPad Software, La Jolla California USA, www.graphpad.com).

### Gut microbiota analysis

Fish were dissected, brain and gut were entirely excised and stored in RNAlater (Ambion, Carlsbad CA, USA) at −80 °C. The detailed description of the aseptic dissection procedure is reported in the Supplementary information (Supplementary methods).

After 30 min lysozyme treatment (50 mg/ml) at 37 °C, DNA was extracted from zebrafish gut (60 mg) using the QIAamp Stool Mini Kit (Qiagen, West Sussex, UK) with minor modifications^44^ and quantified using a Nanodrop TM-1000 Spectrophotometer (Thermo Scientific Ltd, DE, USA).

PCR was conducted to amplify the V3 region of the 16S rRNA gene using the primer pair P3-P2^67^ and the V1-V2 region using the primer pair 27F-338RI/338RII^63^ (Table S2). PCR reactions consisted of 25 μl ReadyMix Taq PCR Reaction Mix with MgCl_2_ (Sigma-Aldrich), 50 pmol of each of primer, 70 ng of DNA in a final volume of 50 μl. Touchdown thermal cycling was conducted using a GeneAmp PCR System 9700 (Perkin-Elmer, CA, USA), applying the conditions previously described^67^.

PCR products (PROBIO n = 5, CTRL n = 5 for each experiment, resulting in a total of ten samples for each group, PROBIO and CTRL, respectively) were purified using the QIAquick PCR Purification Kit (Qiagen). Libraries for sequencing were prepared using the Ion Plus Fragment Library Kit (Life Technologies, USA) and quantified using the Ion Library Quantitation Kit (Life Technologies) and concentrations were adjusted to 26 pM. The emulsion PCR was carried out using the Ion PGM Template OT2 400 Template Kit (Life Technologies) according to the manufacturer’s instructions. Multiplexed sequencing was carried out on a 318 chip using the Ion Torrent PGM system and employing the Ion Sequencing 400 kit (Life Technologies) according to the supplier’s instructions. After sequencing, the sequence reads were filtered and trimmed by the PGM software to remove low quality and polyclonal sequences and quality control was performed using Quantitative Insights Into Microbial Ecology v1.8.0 (QIIME) (quality score >25). In order to calculate downstream diversity measures (alpha and beta diversity indices, UniFrac analysis), 16S rRNA Operational Taxonomic Units (OTUs) were defined at >97% sequence identity. All reads were classified to the lowest possible taxonomic rank using QIIME and a reference dataset from the Ribosomal Database Project. OTUs were assigned using Uclust. The hierarchical clustering based on population profiles of most common and abundant taxa was performed using UPGMA clustering on the distance matrix of OTU abundance. Ecological metrics were calculated on rarefied OTU tables with QIIME to assess sampling depth coverage using observed species, Chao1, Shannon’s diversity index and Good’s coverage. QIIME was also used to calculate Beta diversity metrics among samples using weighted Unifrac distances22 and Bray-Curtis similarity^44^. The distance matrix was represented by a dimensional principal coordinates analysis (PCoA) plots. Taxonomy of intestinal bacterial communities data are presented as means ± standard error of the mean (SEM). Statistical analysis was performed using the two-tailed t test.

### RNA extraction and expression analysis

Total RNA was separately extracted from brain and gut samples using the Pure Link RNA Mini Kit (Ambion, Carlsbad CA, USA) and DNase treatment was performed using the Pure Link DNase Set (Ambion). RNA was quantified using a Nanodrop ND-2000 Spectrophotometer (Thermo Scientific). Total RNA was reverse transcribed using the SuperScript VILO cDNA Synthesis Kit (Ambion) and an oligo dT primer. Expression levels of the *Danio rerio* genes *bdnf*, *tph1a*, *tph1b*, *tph2*, *htr1aa*, *slc6a4a* and *mao* in gut and brain were evaluated by Real Time PCR. PCR amplifications were conducted on 30 ng of first-strand cDNA using the primer pairs listed in Table S3 and the Power SYBR Green Master mix (Ambion). The reactions were conducted in technical triplicates and biological duplicates and were run in the 7500 ABI Thermal Cycler (Applied Biosystems) using the thermal cycle: 50 °C, 2 min; 95 °C, 10 min; 40 cycles of 95 °C, 15 sec and 60 °C, 1 min, followed by a melting curve cycle.

The Real-Time PCR Miner online tool^68^ was used to calculate the PCR efficiency (E) and optimal threshold cycle (C_T_) for each well. The mean relative expression ratio and standard error of the target genes was calculated using the β-actin gene as the endogenous control applying the formula (1 + E target)^–CT target^/(1 + E control)^−CT control^.

Statistical significance of differences in expression levels of the target genes between CTRL and PROBIO groups was assessed by two-tailed t-tests.

## Additional Information

**How to cite this article**: Borrelli, L. *et al*. Probiotic modulation of the microbiota-gut-brain axis and behaviour in zebrafish. *Sci. Rep.* **6**, 30046; doi: 10.1038/srep30046 (2016).

## Supplementary Material

Supplementary Information

Supplementary Video
