# Supplementary material for: Probiotic modulation of the microbiota-gut-brain axis and behaviour in zebrafish
Source: Sci Rep. 2016 Jul 15;6:30046. doi: 10.1038/srep30046 (PMC4945902; doi:10.1038/srep30046)
Supplement: Supplementary Information [file srep30046-s1.pdf]

Probiotic modulation of the microbiota-gut-brain axis and behaviour in zebrafish

Luca Borrelli[a*], Serena Aceto[b*], Claudio Agnisola[b], Sofia De Paolo[b], Ludovico Dipineto[a], Roman M. Stilling[c], Timothy G. Dinan[c,d], John F. Crian[c,e], Lucia F. Menna[a], Alessandro Fioretti[a]

[a] Department of Veterinary Medicine and Animal Productions, University of Naples Federico II, Napoli, Italy

[b] Department of Biology, University of Naples Federico II, Napoli, Italy

[c] APC Microbiome Institute, University College Cork, Cork, Ireland

[d] Department of Psychiatry and Neurobehavioural Science, University College Cork, Cork, Ireland

[e] Department of Anatomy and Neuroscience, University College Cork, Cork, Ireland

* These two authors have equally contributed to the work.

Corresponding author:

Luca Borrelli, via Delpino 1, 80137 Napoli, Italy, luca.borrelli@unina.it, +39 0812536277

Supplementary Methods

Viability test. The viability of the lyophilized *Lactobacillus rhamnosus* IMC 501 (Synbiotec s.r.l., Camerino, Italy) was performed through plate count. For estimation of the growth rate, 1 g of the lyophilized probiotic was inoculated in 100 ml of de Man Rogosa Sharpe (MRS) broth and, after mixing, was incubated at 37˚C for 24 hours. Viable cells were counted by plating serial dilutions of bacterial culture on MRS/agar followed by anaerobic incubation at 37°C for 24 hours, resulting in $1.65x10^{10}$ CFU/g.

Aseptic dissection procedure. Each zebrafish was placed in a sterile Petri dish and its surface was disinfected with 100% Ethanol under a laminar flow hood.

At first, the head was removed with a sterile blade removing the soft tissue from the ventral side of the skull with sterile forceps. Eyes were removed using small spring scissors. Once the skull was opened, the bone was removed from the ventral side of the brain and, at the end, the skin and skull bones were removed from the dorsal side of the brain that was put into a sterile tube containing 1 ml of RNA Later solution and stored at -80°C until use.

The skin and underlying muscle along the belly from the anal fin to the operculum region were cut. Then, the skin and underlying muscle beginning from above the operculum region posteriorly along the side of the fish and down to the anal fin were cut. The skin and underlying muscle from the side of the fish were removed. Once identified, the whole intestine was separate from the rest of the organs and it was put into a sterile tube containing 1 ml of RNA Later solution and stored at -80°C until use. All the operations were performed with sterile instruments (scissors, forceps and surgical blades).

Video legend

Video S1 Difference between the swimming behaviour of probiotic fed (left) and control (right) zebrafish after four weeks of treatment.

Table S1 Results of ANOVA analysis. DF, degrees of freedom; ns, not significant; ***, p<0.001, **, p<0.01, *, p<0.05.

**Average distance (AD)**<br>ANOVA Table			
**Source of Variation**	**F (DFn, DFd)**	**p value**	**p value summary**
Time	F (1, 88) = 55.20	< 0.0001	***
Treatment	F (1, 88) = 2.239	0.1382	ns
Interaction	F (1, 88) = 0.08536	0.7708	ns
**Sidak's multiple comparisons test: Control - Treated**			
	**t**	**DF**	
T0	0.8514	88	ns
T4	1.265	88	ns

**Distance variance (DV)**<br>ANOVA Table			
**Source of Variation**	**F (DFn, DFd)**	**P value**	**P value summary**
Time	F (1, 88) = 71.66	< 0.0001	***
Treatment	F (1, 88) = 9.042	0.0034	**
Interaction	F (1, 88) = 17.79	< 0.0001	***
**Sidak's multiple comparisons test: Control - Treated**			
	**t**	**DF**	
T0	0.8558	88	ns
T4	5.108	88	***

**Nearest distance (ND)**<br>ANOVA Table			
**Source of Variation**	**F (DFn, DFd)**	**P value**	**P value summary**
Time	F (1, 88) = 2.531	0.1152	ns
Treatment	F (1, 88) = 7.948	0.0059	**
Interaction	F (1, 88) = 3.110	0.0813	ns
**Sidak's multiple comparisons test: Control - Treated**			
	**t**	**DF**	
T0	0.7466	88	ns
T4	3.240	88	**

**Shoal size area (OA)**

ANOVA Table			
**Source of Variation**	**F (DFn, DFd)**	**P value**	**P value summary**
Time	F (1, 20) = 0.5958	0.4492	ns
Treatment	F (1, 20) = 6.609	0.0182	*
Interaction	F (1, 20) = 4.423	0.0483	*
**Sidak's multiple comparisons test: Control - Treated**			
	**t**	**DF**	**P value summary**
T0	0.3308	20	ns
T4	3.305	20	**

**Column position (CP)** ANOVA Table			
**Source of Variation**	**F (DFn, DFd)**	**P value**	**P value summary**
Time	F (1, 20) = 522.0	< 0.0001	***
Treatment	F (1, 20) = 302.4	< 0.0001	***
Interaction	F (1, 20) = 377.8	< 0.0001	***
**Sidak's multiple comparisons test: Control - Treated**			
	**t**	**DF**	**P value summary**
T0	1.447	20	ns
T4	26.04	20	***

**Shuttling Frequency (SF)** ANOVA Table			
**Source of Variation**	**F (DFn, DFd)**	**P value**	**P value summary**
Time	F (1, 20) = 27.64	< 0.0001	***
Treatment	F (1, 20) = 26.73	< 0.0001	***
Interaction	F (1, 20) = 7.862	0.0127	*
**Sidak's multiple comparisons test: Control - Treated**			
	**t**	**DF**	**P value summary**
T0	1.673	16	ns
T4	5.638	16	***

Table S2 Differences in microbiota composition between the control (CTRL) and probiotic fed (PROBIO) group of zebrafish. The last column reports the t-test p value. Significant p values (<0.05) are in bold.

**Phylum**	**CTRL1**	**CTRL1**	**CTRL1**	**CTRL1**	**CTRL2**	**CTRL2**	**CTRL2**	**CTRL3**	**CTRL3**	**CTRL3**	**PROBIO1**	**PROBIO1**	**PROBIO1**	**PROBIO1**	**PROBIO2**	**PROBIO2**	**PROBIO3**	**PROBIO3**	**PROBIO3**	**PROBIO3**	**p t-test**
Actinobacteria	0	0	0	0	2	4	7948	1080	34	106	0	0	0	0	6	12	0	0	0	0	0,260639
Bacteroidetes	0	0	0	0	0	0	0	0	0	0	0	0	0	0	0	0	1	2403	19	1889	0,152152
Cyanobacteria	152	218	121	101	118	54	2347	218	1317	162	106	154	131	114	101	146	0	74	83	152	0,134198
Firmicutes	6496	3261	295	240	301	160	8427	272	423	358	562	967	37965	38696	362	954	46173	27526	310	4567	**0,038644**
Fusobacteria	46610	39970	50575	54677	66026	23673	12779	33037	1897	45062	44630	58523	756	3058	53502	674	48881	56091	40913	29370	0,702074
Proteobacteria	4342	40433	7494	669	4742	419	10908	4742	419	10908	2004	4674	176	279	856	22153	4119	9260	1087	4945	0,420368
Verrucomicrobia	0	0	0	0	0	0	0	0	2	0	0	0	0	0	0	0	0	315	0	104	0,209731
**Class**	**CTRL1**	**CTRL1**	**CTRL1**	**CTRL1**	**CTRL2**	**CTRL2**	**CTRL2**	**CTRL3**	**CTRL3**	**CTRL3**	**PROBIO1**	**PROBIO1**	**PROBIO1**	**PROBIO1**	**PROBIO2**	**PROBIO2**	**PROBIO3**	**PROBIO3**	**PROBIO3**	**PROBIO3**	**p t-test**
Bacilli	6496	3261	295	240	278	152	4052	221	369	304	562	967	37965	38696	334	913	46046	27444	298	4542	0,032628
Fusobacteriia	46610	39970	50575	54677	66026	23673	12779	33037	1897	45062	44630	58523	756	3058	53502	674	48881	56091	40913	29370	0,702074
Alphaproteobacteria	1582	191	79	50	40	15	3733	351	1789	130	150	218	63	79	60	59	18	3841	97	1404	0,721783
Betaproteobacteria	460	315	46	22	30	9	6852	38	304	33	83	71	49	97	79	16361	5	1803	25	1396	0,505191
Deltaproteobacteria	78	482	0	0	0	0	0	0	0	0	1	0	0	1	0	0	0	0	0	0	0,25987
Gammaproteobacteria	2222	39445	7369	597	4672	395	323	5084	406	3366	1770	4385	64	102	717	5733	4096	3616	965	2145	0,30359
Clostridia	0	0	0	0	23	8	4375	51	54	54	0	0	0	0	28	41	127	82	12	25	0,342225
Flavobacteriia	0	0	0	0	0	0	0	1080	34	106	0	0	0	0	0	0	1	2403	19	1889	0,32807
Verrucomicrobiae	0	0	0	0	0	0	0	0	2	0	0	0	0	0	0	0	0	315	0	104	0,209731

Table S2 (continued)

**Order**	**CTRL1**	**CTRL1**	**CTRL1**	**CTRL1**	**CTRL2**	**CTRL2**	**CTRL2**	**CTRL3**	**CTRL3**	**CTRL3**	**PROBIO1**	**PROBIO1**	**PROBIO1**	**PROBIO1**	**PROBIO2**	**PROBIO2**	**PROBIO3**	**PROBIO3**	**PROBIO3**	**PROBIO3**	**p t-test**
Streptophyta	152	218	121	101	118	54	2347	132	200	147	106	154	131	114	101	146	0	7	83	132	0,254067
Bacillales	83	145	42	30	44	17	26	0	0	0	41	49	108	119	37	334	0	0	0	0	0,408801
Lactobacillales	6413	3116	253	210	229	133	909	221	369	304	521	918	37857	38577	295	555	46046	27444	298	4542	**0,029665**
Fusobacteriales	46610	39970	50575	54677	66026	23673	12779	33037	1897	45062	44630	58523	756	3058	53502	674	48881	56091	40913	29370	0,702074
Rhizobiales	1292	147	52	30	40	15	3733	208	690	76	124	198	48	55	60	59	8	3189	64	793	0,731049
Burkholderiales	460	315	46	22	30	9	6852	38	304	33	83	71	49	97	79	16361	5	1803	25	1396	0,505191
Desulfovibrionales	78	482	0	0	0	0	0	0	0	0	1	0	0	1	0	0	0	0	0	0	0,25987
Aeromonadales	807	302	7031	408	4669	390	14	4862	244	3295	1658	4257	35	58	712	36	4006	2215	893	1684	0,501784
Enterobacteriales	1221	687	112	34	0	0	0	146	21	29	41	47	19	20	0	0	83	86	61	10	0,162198
Pseudomonadales	144	47	126	140	3	5	309	76	141	42	45	42	1	4	5	5697	7	1315	11	451	0,261791
Vibrionales	50	38409	100	15	0	0	0	0	0	0	26	39	9	20	0	0	0	0	0	0	0,329473
Actinomycetales	0	0	0	0	2	4	7948	0	0	0	0	0	0	0	6	12	0	0	0	0	0,331237
Clostridiales	0	0	0	0	23	8	4375	51	54	54	0	0	0	0	28	41	127	82	12	25	0,342225
Flavobacteriales	0	0	0	0	0	0	0	1080	34	106	0	0	0	0	0	0	1	2403	19	1889	0,32807
Stramenopiles	0	0	0	0	0	0	0	86	1117	15	0	0	0	0	0	0	0	67	0	20	0,322208
Rhodobacterales	0	0	0	0	0	0	0	1	13	2	0	0	0	0	0	0	9	493	6	521	0,1502
Rhodospirillales	0	0	0	0	0	0	0	142	1086	52	0	0	0	0	0	0	1	159	27	90	0,368674
Verrucomicrobiales	0	0	0	0	0	0	0	0	2	0	0	0	0	0	0	0	0	315	0	104	0,209731
Other	290	44	27	20	5	2	3117	0	0	0	26	20	15	24	2	24	0	0	0	0	0,286033

Table S2 (continued)

**Genus**	**CTRL1**	**CTRL1**	**CTRL1**	**CTRL1**	**CTRL2**	**CTRL2**	**CTRL2**	**CTRL3**	**CTRL3**	**CTRL3**	**PROBIO1**	**PROBIO1**	**PROBIO1**	**PROBIO1**	**PROBIO2**	**PROBIO2**	**PROBIO3**	**PROBIO3**	**PROBIO3**	**PROBIO3**	**p t-test**
Bacillus	83	145	42	30	0	0	0	0	0	0	41	49	108	119	0	0	0	0	0	0	0,937725
Lactobacillus	4590	2186	102	99	98	59	38	84	134	97	190	132	20299	18926	121	90	5060	715	62	291	0,156305
Leuconostoc	150	220	112	73	107	65	40	100	178	153	116	279	170	186	99	153	1	9	94	136	0,887507
Streptococcus	303	140	9	5	0	0	0	3	6	11	37	15	3759	4368	0	0	4792	2519	19	643	**0,024902**
Cetobacterium	46588	39960	50554	54662	66005	23663	12779	33037	1897	45062	44617	58505	756	3057	53486	674	48881	56091	40913	29370	0,702385
Bradyrhizobium	811	3	0	1	0	0	0	0	0	0	31	42	3	1	0	0	0	0	0	0	0,375445
Lawsonia	78	482	0	0	0	0	0	0	0	0	1	0	0	1	0	0	0	0	0	0	0,25987
Plesiomonas	1221	687	112	34	0	0	0	146	21	29	41	47	19	20	0	0	83	86	61	10	0,162198
Pseudomonas	144	45	124	125	1	4	2	76	141	42	45	42	1	4	4	4413	7	1315	11	451	0,220921
Vibrio	49	37103	94	15	0	0	0	0	0	0	26	37	9	20	0	0	0	0	0	0	0,329507
Micrococcus	0	0	0	0	0	0	1147	0	0	0	0	0	0	0	1	4	0	0	0	0	0,332625
Mycobacterium	0	0	0	0	1	1	2827	0	0	0	0	0	0	0	3	1	0	0	0	0	0,330861
Propionibacterium	0	0	0	0	1	3	3974	0	0	0	0	0	0	0	2	7	0	0	0	0	0,331106
Staphylococcus	0	0	0	0	0	1	0	0	0	0	0	0	0	0	0	270	0	0	0	0	0,332315
Enterococcus	0	0	0	0	0	0	823	0	0	0	0	0	0	0	0	1	0	0	0	0	0,331138
Pediococcus	0	0	0	0	1	0	0	0	0	0	0	0	0	0	2	293	0	0	0	0	0,328617
Acidaminococcus	0	0	0	0	2	0	4365	0	0	0	0	0	0	0	6	5	0	0	0	0	0,331513
Cupriavidus	0	0	0	0	15	4	9	0	0	0	0	0	0	0	12	16315	0	0	0	0	0,330989
Polynucleobacter	0	0	0	0	5	0	6834	0	0	0	0	0	0	0	1	3	0	0	0	0	0,330458
Acinetobacter	0	0	0	0	2	1	307	0	0	0	0	0	0	0	1	1284	0	0	0	0	0,469653
Flavobacterium	0	0	0	0	0	0	0	1080	34	106	0	0	0	0	0	0	1	2403	19	1889	0,32807
Mesorhizobium	0	0	0	0	0	0	0	13	222	10	0	0	0	0	0	0	7	644	25	356	0,29503
Agrobacterium	0	0	0	0	0	0	0	18	105	32	0	0	0	0	0	0	1	2404	24	301	0,295262
Rhodobacter	0	0	0	0	0	0	0	1	11	2	0	0	0	0	0	0	8	202	4	212	0,150636
Hydrogenophaga	0	0	0	0	0	0	0	15	123	8	0	0	0	0	0	0	2	1051	14	1003	0,178005
Prosthecobacter	0	0	0	0	0	0	0	0	2	0	0	0	0	0	0	0	0	315	0	104	0,209731
Other	2978	2667	7205	559	4801	446	9188	5292	2879	3590	1954	4625	13800	15316	817	207	40305	27691	1122	6069	0,113137

Table S3 Sequence (5’-3’) of the primers used.

Gene	GenBank accession number	Forward	Reverse
β-actin	NM_131031	CACAGATCATGTTCGAGACC	GGTCAGGATCTTCATCAGGT
bdnf	U42489	GCTCAGTCATGGGAGTCC	ATAGTAACGAACAGGATGG
tph1a	AF548566	CAGTTCAGTCAGGAGATTGG	GACAGTGCGTGCTTCAG
tph1b	NM_001001843	TTATATTATTATCTGCCTTGTCTG	AGTGCTCTGTGGTATTTGG
tph2	NM_214795	CAAGAGACAACAGCAACTATG	AAGCCCAACAGGTGATTTAG
htr1aa	EH441641	AGAGCAGCGAGGTGAC	GAGCCGATGATTTGGTAAC
slc6a4a	DQ285098	AGTGGACCTGGGCAATG	AGAAGATACGGCAAGAGAAG
mao	NM_212827	GCAGTCAGAGCCCGAATC	CACACCCATAAACTTGAGGAATC
16S rRNA (V3 region)		CGCCCGCCGCGCGCGGCGGGCGGGGCGGGGGCACGGGGGGCCTACGGGAGGCAGCAG	ATTACCGCGGCTGCTGG
16S rRNA (V1-2 region)		AGAGTTTGATCMTGGCTCAG	GCWGCCTCCCGTAGGAGT
16S rRNA (V1-2 region)		AGAGTTTGATCMTGGCTCAG	GCWGCCACCCGTAGGTGT

Figure 1 Schematic representation of behavioural indexes utilized in the present study. Position of each animal in the tank (frontal projection) is defined by the coordinates (in cm) of the fish eye in a x,y plan whose origin is in the left-bottom corner. D = inter-individual distance; AD = average inter-individual distance; ND = nearest distance; OA = shoal size area; CP = column position, i.e. the vertical distance (in cm) from water surface. During the tracking analysis (with Tracker 4.87), the front plane of the tank was digitally divided into four quadrants: Upper Left (UL), Bottom Left (BL), Upper Right (UR) and Bottom Right (BR). The counting of the total number per minute of the times the fish entered the left, upper, right and left halves of the tank measured the shuttling frequency (SF) of animals and represented the general locomotor activity of the shoal.

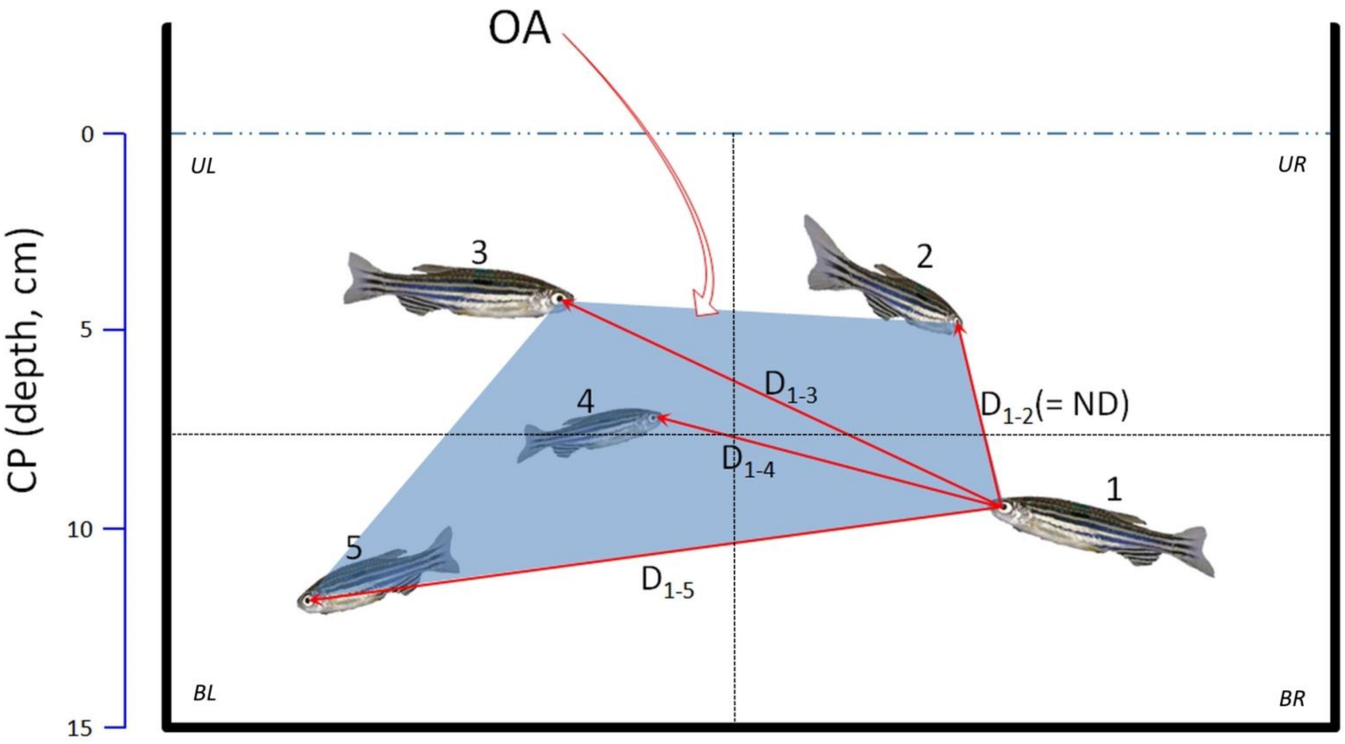

$$AD_1 = \frac{(D_{1-2} + D_{1-3} + D_{1-4} + D_{1-5})}{4}$$

$$DV_1 = \text{standard deviation of } AD_1$$
